# Stacked multi-electrode design of microbial electrolysis cells for rapid and low-sludge treatment of municipal wastewater

**DOI:** 10.1186/s13068-019-1368-0

**Published:** 2019-02-08

**Authors:** Hui Guo, Younggy Kim

**Affiliations:** Department of Civil Engineering, McMaster University, 1280 Main St. W., JHE 301, Hamilton, ON L8S 4L8 Canada

**Keywords:** Microbial electrolysis cells, High electric current, Stacked electrode design, Primary clarifier effluent, Rapid organic removal, Low-sludge wastewater treatment system

## Abstract

**Background:**

Microbial electrolysis cells (MECs) can be used for energy recovery and sludge reduction in wastewater treatment. Electric current density, which represents the rate of wastewater treatment and H_2_ production, is not sufficiently high for practical applications of MECs with real wastewater. Here, a sandwiched electrode-stack design was proposed and examined in a continuous-flow MEC system for more than 100 days to demonstrate enhanced electric current generation with a large number of electrode pairs.

**Results:**

The current density was boosted up to 190 A/m^3^ or 1.4 A/m^2^ with 10 electrode pairs stacked in an MEC fed with primary clarifier effluent from a municipal wastewater treatment plant. High organic loading rate (OLR) resulted in high electric current density. The current density increased from 40 to 190 A/m^3^ when the OLR increased from 0.5–2 kg-COD/m^3^/day to 8–16 kg-COD/m^3^/day. In continuous-flow operation with two stacked MECs in series, the biochemical oxygen demand (BOD) removal was 90 ± 2% and the chemical oxygen demand (COD) removal was 75 ± 9%. In addition, the sludge production was 0.06 g-volatile suspended solids (VSS)/g-COD removed at a hydraulic retention time of only 0.63 h. The electric energy consumption was low at 0.40 kWh/kg-COD removed (0.058 kWh/m^3^-wastewater treated).

**Conclusions:**

The MECs with the stacked electrode design successfully enhanced the electric current generation. The high OLR is important to maintain the high electric current. The organics were removed rapidly and the total suspended solids (TSS) and VSS were reduced substantially in the continuous-flow MEC system. Therefore, the MECs with the stacked electrode design can be used for the rapid and low-sludge treatment of domestic wastewater.

**Electronic supplementary material:**

The online version of this article (10.1186/s13068-019-1368-0) contains supplementary material, which is available to authorized users.

## Background

In conventional wastewater treatment using activated sludge, organic substrates are oxidized in bioreactors by aerobic microorganisms [1]. To maintain aerobic conditions in the bioreactors, oxygen is provided using aeration systems, such as fine bubble diffusers or mechanical aerators [2, 3]. Aeration systems are responsible for a large amount of energy consumption in wastewater treatment. In addition to aeration, return activated sludge pumping also consumes a large amount of electric energy [2, 3]. Therefore, high-energy demand is one of the main challenges in municipal wastewater treatment. Another key challenge in wastewater treatment is the management and final disposal of wastewater sludge that is collected in sedimentation processes. Stabilization of wastewater sludge requires additional processes, such as thickening, anaerobic digestion, and dewatering, and thus makes wastewater treatment expensive and inefficient. In this study, we focused on demonstrating rapid wastewater treatment with minimized biosolids’ production as well as reduced energy consumption.

Microbial electrolysis cells (MECs) can be used for wastewater treatment and simultaneous energy production [4–7]. In an MEC, organic substrates are removed at the bioanode through an oxidation reaction driven by exoelectrogenic bacteria, while hydrogen gas is produced at the cathode by applying a small electric voltage (> 0.13 V) [8–11]. The magnitude of the electric current induced in an MEC represents the rate of the electrode reactions. Thus, the performance of MECs in terms of removing organics in wastewater and producing hydrogen gas is directly dependent on the magnitude of electric current. The current generation in acetate-fed MECs is usually high [12–14] compared to MECs with low acetate concentration. As a result, high electric current (e.g., 40–400 A/m^3^, [12–14]) was feasible, because acetate was used as the primary substrate for exoelectrogenic microorganisms [10]. However, electric current is often low if real wastewater is fed in MECs. It ranged from 7.4 to 42 A/m^3^ [6, 15–19] and increased up to 60 A/m^3^ with Pt catalysts on the cathode [20]. The limited electric current generation, especially with municipal wastewater, indicates that breakthrough improvements are necessary to magnify electric current generation and such improvements should be scalable for pilot-scale and continuous-flow operation for practical wastewater treatment and energy recovery using MECs.

The current generation is proportional to the number of electrode pairs [21] and governed by various other factors, such as organic loading rate, acetate concentration, electrode catalysts, and solution conductivity. In MEC operation using real wastewater, low conductivity is known to be one of the limiting factors for the high current generation. The design of separator electrode assembly was proven to effectively reduce the internal resistance in MEC and thus allow high electric current [22–24]. However, the separator electrode assembly is not ideal for continuous-flow systems, because electrode separator materials can block the flow of wastewater in the MEC reactor. In this study, we modified the separator electrode assembly design using coarse plastic meshes instead of fine separators, such as glass fiber membrane or filter paper. In addition, a cloth-type anode was used to reduce the thickness of the sandwiched electrodes to avoid potential electric short-circuiting. Furthermore, the multi-electrode design was employed in this study to increase the volume-based electric current. In the previous studies, multiple electrodes were applied to improve the COD removal efficiency and electric current generation; however, the total number of electrode pairs was not sufficiently high with 10 anodes and 5 cathodes in a relatively large reactor [25]. In this study, we applied the sandwiched stack design to increase the number of electrode pairs to 10 in a compact MEC reactor, allowing substantially high electric current densities. Although this study examined MECs with 5 and 10 electrode pairs, additional electrode pairs can be added in the modulated MEC design.

In practical wastewater treatment, MECs are expected to produce a much smaller amount of waste sludge than conventional activated sludge because of the small yield coefficient (0.02 g-VSS/g-COD) of exoelectrogenic microorganisms [26, 27]. In this study, we also focused on estimating biosolids production in the newly designed MEC reactors fed with real wastewater. Other specific objectives of this study are to: demonstrate high electric current generation in MECs continuously fed with real wastewater by using sandwiched electrode stacks; investigate the effects of various reactor designs and operation factors, such as wastewater flow rate and organic loading rate on electric current density; examine the wastewater treatability in terms of the rate of organic removal and biosolids production; and compare the MEC performance with conventional activated sludge regarding energy consumption for the treatment of primary clarifier effluent. It should be emphasized that the main novelty of this study is the lab-scale demonstration of low-sludge municipal wastewater treatment using the easily scalable electrode-stack MEC.

## Results and discussion

### Rapid wastewater treatment with high electric current generation

**Fig. 1:**
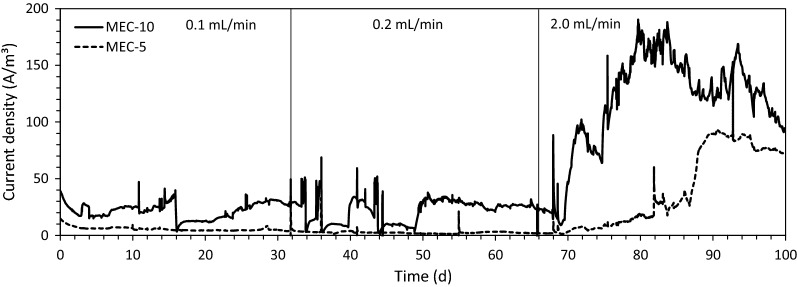
Electric current generation in the continuous-flow MEC system fed with primary clarifier effluent

The multi-electrode MEC showed substantially enhanced electric current generation as the maximum current density was 190 A/m^3^ or 1.4 A/m^2^ in MEC-10 (MEC of 10 electrode pairs) with continuously fed real wastewater (Fig. 1). The maximum current density in MEC-1 (MEC of 1 electrode pair) was only ~ 4 A/m^3^ or 0.2 A/m^2^, which was much smaller than that in MEC-10. This result indicates that a large number of electrode pairs in the sandwiched design can significantly enhance the rate of wastewater treatment using MECs without any precious metal catalysts on the cathode, such as platinum. The current density in MEC-5 (MEC of 5 electrode pairs) was sensitively affected by the flow rate of wastewater and varied in a wide range up to 92 A/m^3^ or 1.2 A/m^2^ (Fig. 1). Note that the primary clarifier effluent was fed to MEC-10 and the effluent from MEC-10 was introduced to MEC-5 during the continuous MEC operation. The low current density at the low flow rates can be explained by low organic substrate concentration in the effluent from MEC-10. For instance, the measured COD concentration in the MEC-10 effluent (i.e., influent to MEC-5) was 10.4 mg/L, while it was 10.0 mg/L in the MEC-5 effluent at 0.2 mL/min. This negligible COD removal in MEC-5 indicates that the wastewater was sufficiently treated in MEC-10, and thus, MEC-5 did not generate high electric current for 0.2 mL/min (and 0.1 mL/min) due to the low COD concentration. In the previous MEC studies conducted with municipal wastewater, the reported electric current density ranging from 11 to 42 A/m^3^ [15–17] is much lower than the current density result in this study. This comparison confirms that the stacked electrode design allowed sufficiently high current generation with primary clarifier effluent. The high current generation can be explained by the significantly high anode surface area (136 m^2^/m^3^ for MEC-10,77 m^2^/m^3^ for MEC-5), enhancing the rate of the electrode reactions in the small MEC reactors.

It should be emphasized that the mean hydraulic residence time (HRT) in the MEC system was 0.63 h at 2 mL/min. Considering the typical residence time of 6–8 h in aeration tanks of conventional activated sludge systems [1, 2], the new MEC stack design can reduce the size of wastewater treatment reactors to approximately 10% of typical aeration tanks in conventional activated sludge for municipal wastewater treatment.

### Organic loading rate and electric current

**Fig. 2:**
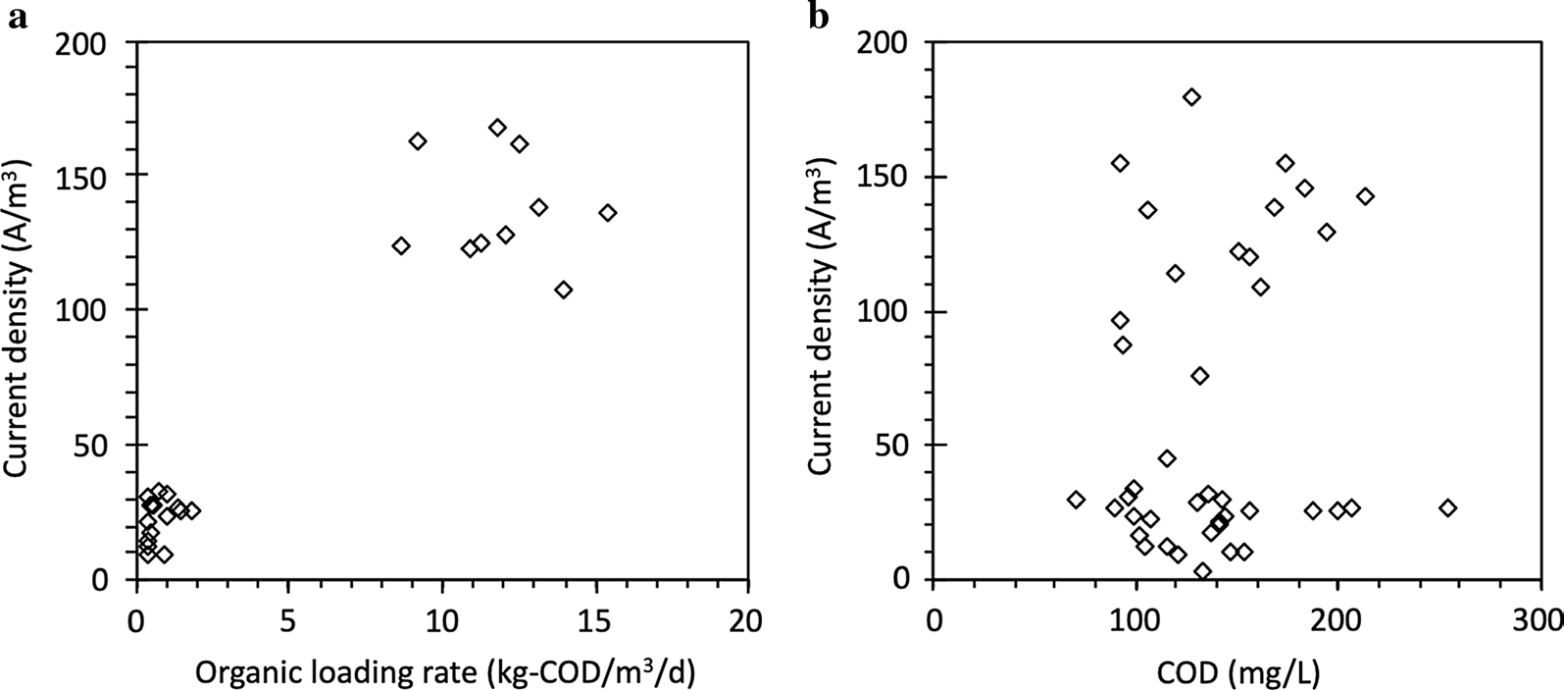
Electric current density in MEC-10 vs. **a** organic loading rate; **b** COD

**Fig. 3:**
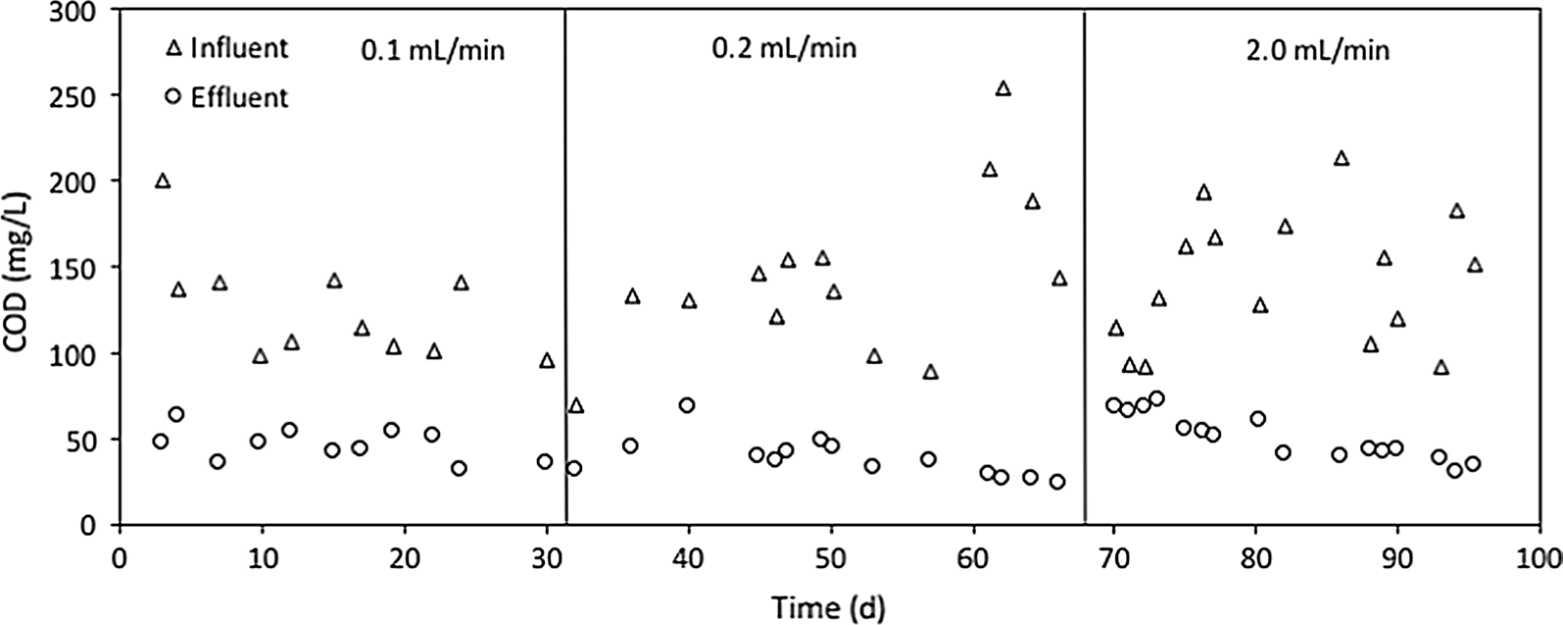
COD concentration of the influent and effluent

The current density was governed by the organic loading rate (OLR) rather than the COD concentration as the high OLR resulted in high electric current density (Fig. 2a). However, the electric current density was not clearly correlated with the COD concentration (Fig. 2b). This finding is consistent with the previous report that the magnitude of electric current is governed by the OLR rather than other operation factors, such as substrate concentration and conductivity, especially when real wastewater is used as the feed for MEC operation [28]. Even with significant variations in the influent COD concentration, the OLR was governed by the flow rate, because the influent COD was below 200 mg/L (Fig. 3). For the slow flow rates (0.1 and 0.2 mL/min), the OLR was smaller than 2 kg-COD/m^3^/day (0.4–0.5 kg-COD/m^3^/day at 0.1 mL/min and 0.7–1.8 kg-COD/m^3^/day at 0.2 mL/min), resulting in low current densities below 40 A/m^3^ (Fig. 2a). In addition, there was no significant increase in the current densities when the flow rate increased from 0.1 to 0.2 mL/min. However, for the high flow rate of 2 mL/min, the current density increased up to 190 A/m^3^ with the relatively high OLR between 8 and 16 kg-COD/m^3^/day (Fig. 2a). The OLR for the high flow rate was much greater than the typical OLR in conventional activated sludge systems (usually smaller than 2 kg-COD/m^3^/day) [2, 29], indicating that the stacked MEC design can treat clarified domestic wastewater more efficiently than conventional activated sludge systems. The relatively short hydraulic residence time in MEC-10 (0.33 h at 2.0 mL/min) did not negatively affect the electric current generation in MEC-10. This finding indicates that exoelectrogenic bacteria can rapidly utilize organic substrates in municipal wastewater, allowing very short hydraulic retention time (i.e., 0.33 h) and thus small MEC reactors for wastewater treatment.

It should be noted that a gradual decrease was observed in the electric current of MEC-10 and MEC-5 between 83 and 93 days (Fig. 1). This result can be explained by decreased OLR for the MEC operation. From 83 to 93 days, the OLR decreased from 15.4 to 6.6 kg-COD/m^3^/day when the COD concentration of the influent decreased from 214 to 92 mg/L simultaneously (Fig. 3).

### Wastewater treatability on organic removal

**Fig. 4:**
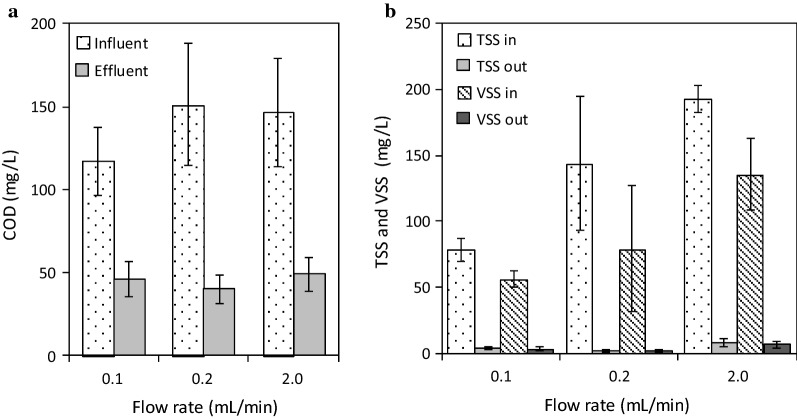
Wastewater treatability of the MEC system: **a** COD removal; **b** TSS and VSS removal

The rate of organic removal was rapid since 75 ± 9% of COD was removed in MEC-10 and MEC-5 for 2 mL/min or the hydraulic residence time (HRT) of 0.63 h. The COD removal was similar or slightly lower at 64 ± 15% and 59 ± 11% for the longer HRT conditions of 12.5 h (0.1 mL/min) and 6.3 h (0.2 mL/min), respectively (Fig. 4a). The average current density was 106.4 A/m^3^ (MEC-10) and 35.0 A/m^3^ (MEC-5) at 2 mL/min, while it was around 23 A/m^3^ (MEC-10) and 3 A/m^3^ (MEC-5) at low flow rates (0.1 mL/min and 0.2 mL/min) (Fig. 1). The electric current in MECs represents the rate of the electrode reactions, which are the organic removal at the anode and hydrogen production at the cathode. Therefore, the higher electric current density contributes to the faster organic removal. In conventional activated sludge systems, the typical HRT in the aeration tank is 6 to 8 h with approximate 80% COD removal [2]. Considering the rapid COD removal of the examined MEC system (75% COD removal in 0.63 h), the stacked MEC design can be used for effective treatment of municipal wastewater. The BOD removal was high at 90 ± 2% with the effluent BOD of 19.4 ± 4.1 mg/L at 2 mL/min. Note that, for real wastewater without its chemical composition, the BOD-based removal efficiency provides more reliable organic removal treatability for biological wastewater treatment [1].

### Wastewater treatability on biosolids reduction

TSS (total suspended solids) and VSS (volatile suspended solids) were reduced substantially in the continuous-flow MEC system. Nearly, complete reduction of biosolids (> 94%) for both VSS and TSS was observed regardless of the flow rate conditions (Fig. 4b). This observed biosolids removal was much higher than 60% of TSS removal reported in single chamber MEC reactors (250 mL) that were fed with domestic wastewater and the MECs were built with only 1 electrode pair [27]. This comparison indicates that the stacked electrode design is beneficial to minimizing sludge generation. The apparent yield coefficient measured in this study ranged from 0.01 to 0.06 g-VSS/g-COD, which is an order of magnitude smaller than the yield coefficient of the other anaerobic microorganisms (typical 0.1–0.6 g-VSS/g-COD) [1, 30, 31]. In this study, the concentration of biosolids in the effluent was 3.67 ± 1.25 mg-TSS/L or 2.02 ± 1.21 mg-VSS/L at 0.1 mL/min, 2.02 ± 1.21 mg-TSS/L or 1.60 ± 0.90 mg-VSS/L at 0.2 mL/min, and 10.36 ± 8.06 mg-TSS/L or 6.4 ± 2.76 mg-VSS/L at 2 mL/min (Fig. 4b). These consistently low VSS and TSS concentrations imply that the MEC effluent can be discharged even without secondary clarification. In addition, the enhanced reduction of biosolids clearly indicates that MECs can dramatically reduce the sludge production in wastewater treatment as well as the cost for sludge treatment and disposal.

### Coulombic efficiency and energy consumption

**Fig. 5:**
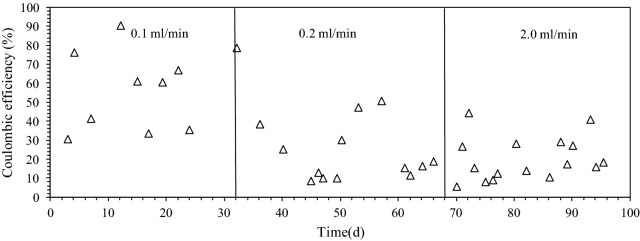
Coulombic efficiency during the continuous-flow MEC system operation

Coulombic efficiency (CE) varied in a wide range (Fig. 5). The wide variation of CE can be explained by the use of real wastewater, where its composition, especially the readily biodegradable portion of COD, changes continuously. A certain degree of variations in CE has been commonly found in the previous studies, where the primary clarifier effluent from wastewater treatment plants was fed in bioelectrochemical systems [15, 16, 32]. The CE was 66 ± 27% at 0.1 mL/min, while it dropped to 22 ± 14% at 0.2 mL/min and 20 ± 11% at 2 mL/min (Fig. 5). A significant decrease was shown in CE when the flow rate increased from 0.1 to 0.2 mL/min. The significant decrease was due to the increase in ∆COD (Fig. 4a) and the similar electric current generation at 0.2 mL/min. The CE value was much lower than the CE of our previous study (> 72%), where the reactor was fed with synthetic wastewater [21]. The low CE results indicate that other biological reactions in the MEC reactors can affect CE. When the MEC reactors were disassembled, thick biofilms were found both on the anode and cathode (Additional file 1: Figure S1), indicating that there was a substantial amount of microorganisms in the system. The observed biomass on the electrodes did not affect the sludge production during the MEC operation, because the sludge production was consistently low over the course of 3-month operation. The presence of other terminal electron acceptors such as ferric iron and sulfate can contribute to the COD removal without electric current generation in MECs [16]. In the ICP-OES (inductive coupled plasma-optical emission spectrometry) analysis, the dissolved iron was detected in the feed wastewater, because the local wastewater treatment plant applied ferric sulfate in the preliminary treatment for phosphorus removal. Note that many of the known exoelectrogenic bacteria (*Geobacter* and *Shewanella* spp.) can utilize iron as the terminal electron acceptor [33, 34]. In addition, other iron-reducing bacteria are commonly found in domestic sewer systems [34]. However, the concentration of iron in the influent and effluent decreased from 1.45 to 0.19 mg/L, which only results in the 0.54 mg/L COD removal. Thus, the effects of iron ions were negligible. Sulfate can also contribute to additional COD removal without electric current generation by sulfate reducing bacteria [35]. Sulfate concentration in the wastewater was consistently high at 82–122 mg/L [36], which can potentially oxidize up to 81 mg/L of COD assuming H_2_S production. In addition, the methane production in MEC can also result in the low CE.

The electric energy consumption was as low as 0.40 kWh/kg-COD removed or 0.058 kWh/m^3^ wastewater treated (Additional file 2: Figure S2). The low energy consumption for the MEC operation can be explained by the stacked electrode design with the reduced inter-electrode distance (2.8 mm) and significantly magnified electrode surface area with a total of 15 electrode pairs sandwiched in the small reactors. The short inter-electrode distance contributed to the low inter resistance (229.5 Ω cm^2^ based on 1.22 mS/cm and 2.8 mm) and thus resulted in the low-voltage drop between the electrodes (3 to 18 mV). Compared to the energy consumption of conventional activated sludge systems which typically ranged from 0.7 to 2 kWh/kg-COD removed [2], the energy consumption for MEC operation was much lower. In conclusion, the stacked multi-electrode MECs can replace the activated sludge system because of the great treatability of primary clarifier effluent and low energy consumption.

Note that the energy recovered, as H_2_ gas production was not included in the energy requirement calculation, because biogas production, including H_2_ gas, was very small. The small biogas production can be explained by short hydraulic residence time in the MEC reactor (37.5 min at 2 mL/min). For the short residence time, tiny H_2_ gas bubbles from the cathode were flowing with the wastewater rather than separated by gravity in the MEC reactors. In addition, H_2_ gas is rapidly converted into CH_4_ in MECs without proper inhibition of hydrogenotrophic methanogens. According to the Henry’s law constant (769 atm/M) [37], the examined highest flow rate can carry more methane (606 mg CH_4_/day) than the maximum amount of methane that can be produced in the MEC (155 mg CH_4_/day; 100% conversion of H_2_ into CH_4_; 100% H_2_ production from electric current).

### Individual electrode performance

During the continuous-flow operation, the wastewater flowed through 10 independent electrode pairs serially in MEC-10 and then 5 electrode pairs in MEC-5. The electric current generation was similar among the multiple electrode pairs (Table 1), and this result was not consistent with our recent study with synthetic wastewater that is considered to have resulted in a significant variation in the electric current generation among the electrode pairs [21]. The different performances of the reactor with real wastewater and synthetic wastewater were due to the low readily biodegradable organics concentration of real wastewater. The slow conversion of biodegradable organics to readily biodegradable organics in the reactor contributes to the stable electric current results. In addition, the consistent electric current results indicate that the operation of the MEC stack was reliable even though a single reactor was operated. The location of individual electrode pairs did not affect the current generation except for the first pair from the inlet (electrode pair #1). For instance, there was no significant variation in the electric current for electrode pairs #2 through #10 in MEC-10 (Table 1). However, electrode pair #1 generated noticeably lower electric current compared to the other electrode pairs only at the high flow rate of 2 mL/min (Table 1).

**Table 1:** Average electric current (mA) for each electrode pair in MEC-10

Electrode pair	0.1 mL/min	0.2 mL/min	2.0 mL/min
#1	0.11 ± 0.09	0.08 ± 0.03	0.15 ± 0.08
#2	0.14 ± 0.05	0.09 ± 0.05	0.53 ± 0.19
#3	0.12 ± 0.05	0.10 ± 0.06	0.50 ± 0.15
#4	0.08 ± 0.03	0.10 ± 0.04	0.54 ± 0.17
#5	0.08 ± 0.03	0.09 ± 0.04	0.54 ± 0.18
#6	0.08 ± 0.03	0.09 ± 0.05	0.55 ± 0.20
#7	0.08 ± 0.03	0.11 ± 0.04	0.56 ± 0.20
#8	0.08 ± 0.03	0.09 ± 0.04	0.47 ± 0.17
#9	0.07 ± 0.02	0.09 ± 0.04	0.49 ± 0.18
#10	0.07 ± 0.03	0.08 ± 0.04	0.53 ± 0.22

### Conclusions and outlook

The stacked electrode design demonstrates the excellent wastewater treatability with the minimal biosolids production and consistently low COD in the MEC effluent. Even though the demonstration was achieved in lab-scale experiments, the MEC design is readily applicable in practical applications, as the experiment was conducted with primary clarifier effluent from a local wastewater treatment plant. They demonstrated that MEC design does not need further scale-up for practical applications. Many MEC reactors with 10–20 electrode pairs can be used to receive the primary clarifier effluent in parallel just like a modulated membrane filtration system, where individual membrane modules receive feed water in parallel and operate independently one another. Without further scale-up of the MEC design, the stacked electrode MECs can be used to treat municipal wastewater with stable effluent quality and minimal biosolids’ generation.

## Methods

### Stacked MEC construction and start-up

**Fig. 6:**
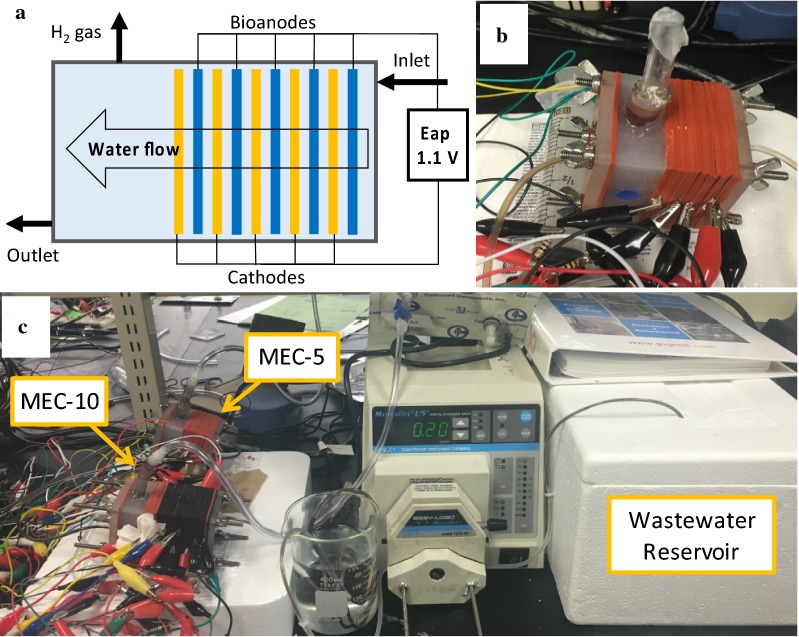
**a** Schematic of the sandwiched electrode stack; **b** MEC-5; **c** continuous-flow system with MEC-10 and MEC-5 in series

Three MEC reactors were built using polypropylene blocks by drilling a cylindrical hole (7 cm^2^ in cross section). The structure of these reactors is the same as the structure of our previous study [21]. The liquid volumes of MEC-10 (10 electrode pairs), MEC-5 (5 electrode pairs), and MEC-1 (1 electrode pair) were 40, 35, and 28 mL, respectively. The anode was activated carbon cloth (ACC100, Evertech Envisafe Ecology, Taiwan) and pretreated in a surfactant solution [38]. The cathode was stainless steel mesh (304 stainless steel, 200 × 200 mesh, McMaster Carr, USA) without any precious metal catalysts. Each electrode pair consisted of one carbon cloth piece and one stainless steel mesh piece, which were sandwiched and separated using two rubber gaskets and one plastic mesh. The thickness of the two gaskets (i.e., the distance between the anode and cathode) was 2.8 mm. Each electrode pair was operated independently (i.e., no electrical connections between the electrode pairs). The feed wastewater flowed in the normal direction to the sandwiched electrodes (Fig. 6a). To ensure proper wastewater flow and biogas collection through the stacked electrode pairs, the upper and lower parts of each electrode (both the anode and cathode) were cut off by 0.3 cm from the circular edge (Additional file 1: Figure S1), resulting in 5.42 cm^2^ of the total surface area per electrode. A plastic tube was placed on the top of the polypropylene block for biogas collection (Fig. 6b). The constructed MECs were inoculated using effluent from existing MECs and operated using synthetic wastewater for approximately 3 months (1 g/L sodium acetate in 20 mM PBS buffer with vitamins and minerals [21, 39]), before the main part of the experiment was conducted with real wastewater.

### MEC operation

The feed wastewater (primary clarifier effluent) was collected from a local wastewater treatment plant (Woodward Wastewater Treatment Plant, Hamilton, ON, Canada). The collected wastewater was used immediately in the experiment or stored at 4 °C for no longer than 7 days. The quality parameters of the primary clarifier were measured before feeding into the systems. MEC-10 and MEC-5 were serially arranged where the wastewater flowed through MEC-10 and then MEC-5 to achieve the maximum treatability of our MEC reactors. The MEC system was operated in the continuous-flow mode with three different flow rates (0.1, 0.2, and 2.0 mL/min). It was operated for 32 days at 0.1 mL/min, 36 days at 0.2 mL/min, and 32 days at 2.0 mL/min. The feed wastewater reservoir was kept in an icebox and refilled with fresh wastewater every day under the low flow rate conditions (0.1 and 0.2 mL/min) and filled twice a day at the high flow rate (2 mL/min). Between the reservoir and MEC-10, a 30-cm long copper tube was submerged in a beaker filled with water to equilibrate the influent wastewater temperature to the room temperature (22.2 ± 0.7 °C) (Fig. 6c). MEC-1 with a single electrode pair was operated in a fed-batch mode using the same primary clarifier effluent but independently from the continuous operation of MEC-10 and MEC-5.

The applied voltage (*E*_ap_) to each of the electrode pairs was 1.1 V using an external power supplier (GPS-1850D; GW Instek, Taiwan). The electric current for each electrode pair was determined by measuring the electric voltage across a 10-Ω external resistor. A digital multimeter and data acquisition system were used to record the voltage every 20 min (Model 2700, Keithley Instruments, OH). The electric current for individual electrode pairs was added for all electrode pairs in the reactor and then normalized by the effective volume of the MEC reactor to obtain volume-based current density. In addition to the volume-based current density, the area-based current density was also provided using the total anode surface area of the MEC reactors (54.2 cm^2^ for MEC-10 and 27.1 cm^2^ for MEC-5).

### Experimental analysis

The influent and effluent of the serial MEC system were collected every weekday and analyzed for total suspended solids (TSS), volatile suspended solids (VSS), chemical oxygen demand (COD), and biochemical oxygen demand (BOD) in accordance with the standard method [40]. The COD analysis was conducted using commercial COD test tubes (Method 8000u, Hach Company, USA).

The wastewater treatment plant applied ferric sulfate for phosphorus removal. The high concentration of ferric ions can be used as the electron acceptors and further contributes to the COD removal in MECs. Therefore, the influent and effluent samples were also analyzed to quantify ferric iron in the wastewater. The collected sample (4.5 mL) was immediately acidified with 1.5 mL 98% sulfuric acid (v/v) and filtered using a syringe filter (pore size 0.45 μm, polyethersulfone membrane, VWR International, USA). The filtered sample was analyzed in ICP-OES (Vista Pro, Varian Inc., Australia) to determine iron concentration.

The wastewater influent and effluent were also analyzed for pH and conductivity (SevenMulti, Mettler-Toledo International Inc., USA). In MEC-10 and MEC-5, pH of the wastewater was slightly increased from 6.7 ± 0.3 (influent) to 7.3 ± 0.2 (effluent), since the hydroxide ions were produced at the cathode with hydrogen production. The wastewater conductivity was 1.22 ± 0.15 mS/cm and did not change in the MEC experiment. No additional pre-treatment or modification was conducted to improve the wastewater treatability.

### Coulombic efficiency and energy consumption

The Coulombic efficiency (CE) is the electron-based ratio between the amount of electric current generated and COD removed [41]:

1 $${\text{CE}} = \frac{8I}{{QF\Delta {\text{COD}}}}$$

where *I* is the summation of the electric current for all electrode pairs in the MEC, *F* is the Faraday’s constant (96,485 C/mol), and ΔCOD is the COD change between the influent and effluent, and *Q* is the flow rate of wastewater.

The COD-based energy consumption (*W*_COD_) in kWh/kg-COD removed is calculated by dividing the electric energy consumption by the rate of COD removal as

2 $$W_{\text{COD}} = \frac{{E_{\text{ap}} I}}{{ Q\Delta {\text{COD}}}}.$$

The volume-based energy consumption (*W*_v_) in kWh/m^3^ of treated wastewater is calculated by dividing the total energy consumption by the flow rate during the experiment as

3 $$W_{\text{v}} = \frac{{E_{\text{ap}} I}}{ Q}.$$

## Additional files

**Additional file 1: Figure S1.** (a) cathode and (b) bioanode after ~ 3 months of MEC operation with primary clarifier effluent.

**Additional file 2: Figure S2.** Electric energy consumption for continuous-flow MEC system operation.

## Abbreviations

BOD: biochemical oxygen demand
CE: coulombic efficiency
COD: chemical oxygen demand
*E*_ap_: applied voltage (V)
HRT: hydraulic residence time
ICP-OES: inductive-coupled plasma optical emission spectrometry
MEC: microbial electrolysis cell
MEC-1: MEC with 1 electrode pair
MEC-5: MEC with 5 electrode pairs
MEC-10: MEC with 10 electrode pairs
OLR: organic loading rate
TSS: total suspended solids
VSS: volatile suspended solids
*W*_COD_: COD-based energy consumption (kWh/kg-COD removed)
*W*_v_: volume-based energy consumption (kWh/m^3^ of treated wastewater)
ΔCOD: COD change between the influent and effluent (mg/L)
