# Supplementary material for: Stacked multi-electrode design of microbial electrolysis cells for rapid and low-sludge treatment of municipal wastewater
Source: Biotechnol Biofuels. 2019 Feb 8;12:23. doi: 10.1186/s13068-019-1368-0 (PMC6367776; doi:10.1186/s13068-019-1368-0)
Supplement: Supplementary file 1 — Additional file 1: Figure S1. (a) cathode and (b) bioanode after ~ 3 months of MEC operation with primary clarifier effluent. [file 13068_2019_1368_MOESM1_ESM.docx]

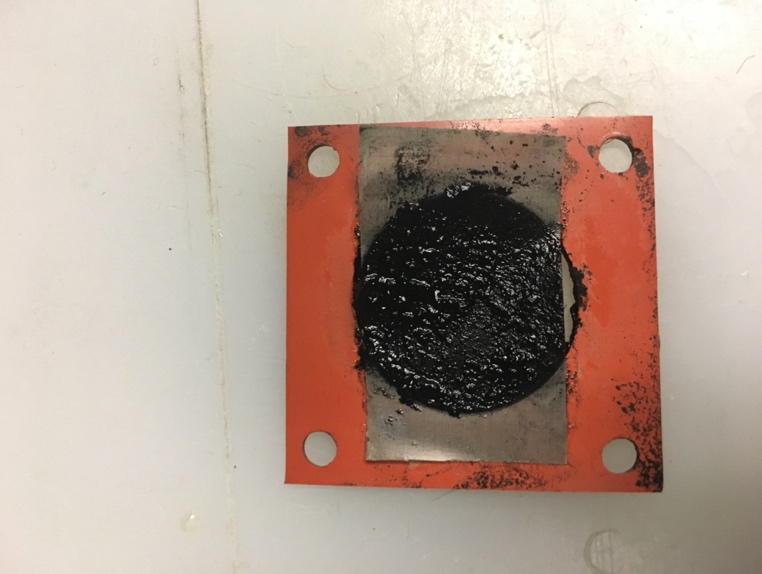

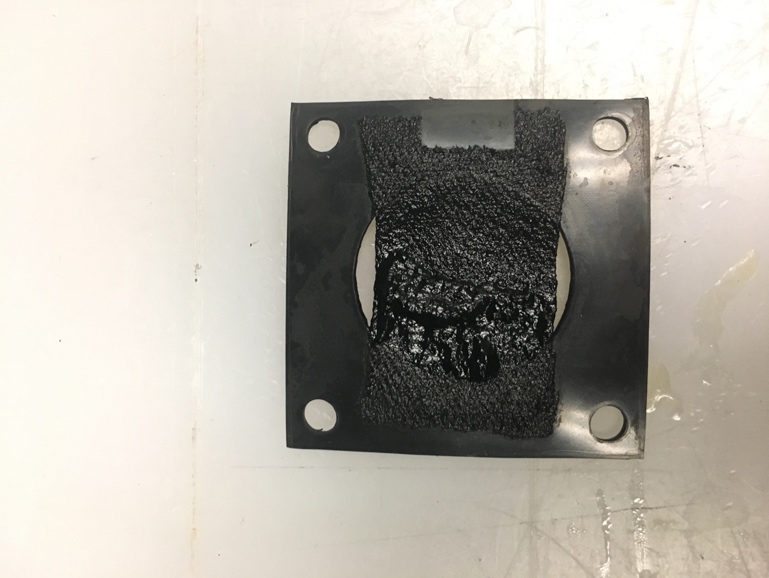


(b)

(a)

**Figure S1.** (a) Cathode and (b) bioanode after ~3 months of MEC operation with primary clarifier effluent.
