# Supplementary material for: Stacked multi-electrode design of microbial electrolysis cells for rapid and low-sludge treatment of municipal wastewater
Source: Biotechnol Biofuels. 2019 Feb 8;12:23. doi: 10.1186/s13068-019-1368-0 (PMC6367776; doi:10.1186/s13068-019-1368-0)
Supplement: Supplementary file 2 — Additional file 2: Figure S2. Electric energy consumption for continuous-flow MEC system operation. [file 13068_2019_1368_MOESM2_ESM.docx]

**Figure S2.** Electric energy consumption for continuous-flow MEC system operation.
